# Protocol for a feasibility randomised controlled study of a multicomponent intervention to promote a sustainable return to work of workers on long-term sick leave — PROWORK: PROmoting a Sustainable and Healthy Return to WORK

**DOI:** 10.1186/s40814-022-01143-8

**Published:** 2022-08-19

**Authors:** Veronica Varela-Mato, Kate Godfree, Anwar Adem, Holly Blake, Craig Bartle, Guy Daly, Juliet Hassard, Richard Kneller, Caroline Meyer, Sean Russell, Steven Marwaha, Charlotte Kershaw, Kristina Newman, Joanna Yarker, Louise Thomson, Fehmidah Munir

**Affiliations:** 1 School of Sport, Exercise and Health Sciences, Loughborough University, Loughborough, UK; 2 School of Economics, University of Nottingham, Nottingham, UK; 3 School of Health Sciences, Queen’s Medical Centre, University of Nottingham, Nottingham, UK; 4 NIHR Nottingham Biomedical Research Unit, Nottingham, UK; 5 Institute of Mental Health, Nottinghamshire NHS Healthcare Trust, Nottingham, NG3 6AA UK; 6 Faculty of Health and Life Sciences, Coventry University, Coventry, UK; 7 Office of the Provost, The British University in Egypt, El Sherouk City, Cairo 11837 Egypt; 8 School of Medicine, University of Nottingham Jubilee Campus, Nottingham, NG8 1BB UK; 9 Executive Office, Warwick University, Coventry, CV4 7AL UK; 10 Institute for Mental Health, School of Psychology, University of Birmingham, Birmingham, UK; 11 Warwick Manufacturing Group, University of Warwick, Coventry, UK; 12 Psychology Department, Nottingham Trent University, Nottingham, UK; 13 Affinity Health at Work and Birkbeck, University of London, London, UK

**Keywords:** Return to work, Long-term sickness absence, Mental health, Small and medium enterprises, Large enterprises, Positive communication, Worker, Manager, Intervention

## Abstract

**Background:**

The cost of sickness absence has major social, psychological and financial implications for individuals and organisations. Return-to-work (RTW) interventions that support good quality communication and contact with the workplace can reduce the length of sickness absence by between 15 and 30 days. However, initiatives promoting a sustainable return to work for workers with poor mental health on long-term sickness absence across small, medium and large enterprises (SMEs and LEs) are limited. This paper describes the protocol of a pilot randomised controlled trial (RCT) to test the feasibility of implementing a RTW intervention across SMEs and LEs across all sectors.

**Methods and design:**

A two-arm feasibility RCT with a 4-month intervention will be conducted in SMEs and LE enterprises from the Midlands region, UK. At least 8 organisations (4 controls and interventions), and at least 60 workers and/or managers, will be recruited and randomised into the intervention and control group (30 interventions, 30 controls). Workers on long-term sickness absence (LTSA) (between 8 and 50 days) and managers with a worker on LTSA will be eligible to participate. The intervention is a behavioural change programme, including a managers and workers RTW toolkit, focused on supporting sickness absence and RTW through the provision of knowledge, problem-solving, action planning, goal setting and positive communication that leads to a sustainable RTW. Organisations assigned to the control group will continue with their usual practice. Measurements of mental health, RTW, work outcomes, quality-of-life, workplace support and communication and other demographic data will be taken at baseline, 2 months and 4 months. Feasibility will be assessed based on recruitment, retention, attrition, completion of measures and intervention compliance for which specific process and research outcomes have been established. A process evaluation will explore the experiences and acceptability of the intervention components and evaluation measures. Exploratory economic evaluation will be conducted to further inform a definitive trial.

**Discussion:**

This is a novel intervention using a worker-manager approach to promote a sustainable return to work of workers on long-term sick leave due to poor mental wellbeing. If this intervention is shown to be feasible, the outcomes will inform a larger scale randomised control trial.

**Trial registration:**

ISRCTN90032009 (retrospectively registered, date registered 15th December 2020)

## Background

In Britain, mental health issues increasingly account for a significant proportion of long-term sickness absence from work [1], and those who are absent for 6 months or longer have less than a 50% chance of ever returning to employment [2]. Sickness absence associated with poor mental health costs UK employers £7 billion each year [1], and a recent report by the Office for National Statistics (2019) [3] suggests sickness absence due to poor mental health is on the rise (12.4% in 2018 compared to 9.1% in 2009). The cost of sickness absence is not at an economic level only, as there are major social, psychological and financial implications for individuals on sick leave and for those unable to RTW [4]. These include inactivity, isolation, reduced workability and productivity [5–8], with reduced wellbeing and impaired self-image leading to the individual potentially withdrawing from society and support networks [5]. Long-sickness absence is also a predictor of disability pension, higher risk of unemployment and job termination [9]. Therefore, early intervention to support a worker’s RTW is not only cost-effective for the employer (e.g. in terms of reduced turnover, recruitment costs, retention of knowledge) but also is vital for workers’ health and wellbeing.

A systematic review and meta-analysis of randomised controlled trials (RCT) and cluster RCTs published by Nigatu and colleagues (2016) [10] found that RTW interventions providing regular contact and communication with the individual’s workplace alongside health interventions based on cognitive behavioural therapy or a problem-solving approach were effective in reducing the number of sick leave days (−13.38 days) in individuals with a common health problem. These findings are supported by a more recent systematic review and meta-analysis led by Mikkelsen (2018) [11], who observed an average reduction in time until RTW of 15–30 days, and by Nieuwenhuijsen and colleagues (2020) [12] who reported in their Cochrane review that a combination of work-directed and clinical interventions (such as psychological treatment) reduces sickness absence days within the first year of follow-up (*SMD* −0.25, 95% *CI* −0.38 to −0.12: 9 studies). These studies support the economic value of investing in evidence-based RTW interventions. However, in the UK, the RTW of workers on long-term sick leave is usually managed by employers with the responsibility given to line managers, particularly in small- and medium-sized organisations. In some cases, employers may outsource the RTW support to external occupational health providers. Recommendations from the National Institute for Health and Care Excellence (NICE) (2019) [13] indicate that organisations should develop policies and procedures that promote health and wellbeing and a sustainable RTW. Yet, information regarding specific actions of how to achieve it is limited. The NICE guidelines (2019) [13] further recommend for employers to make early and positive contact with their workers on long-term sick leave to make them feel supported, valued and confident about returning to work. Early communication may also prevent the development of poor mental health as a comorbidity in workers on long-term sickness due to other health conditions [14].

The outbreak of coronavirus SARS-2 (COVID-19), declared a pandemic by the World Health Organization (WHO) in March 2020, presents a challenging scenario for a safe RTW following long-term sick leave, particularly amongst those workers with poor mental health and wellbeing. Since early in 2020, drastic changes to workplaces have been implemented to ensure the safety of all their workers, which means that some workers will have to RTW remotely following a period of sick leave or may experience additional anxiety towards RTW on site. Small- and medium-sized enterprises (SMEs) may have limited resources to support these workers, and they are also less likely to have formal policies and procedures in place to keep in touch with people on sickness absence. Therefore, initiatives promoting a supportive and sustainable RTW experience for workers with poor mental health are now more important than ever and particularly amongst SMEs (with 10 to 249 workers). In the UK, these account for 99.9% of the business population (5.9 million businesses) [15].

Based on recent evidence and the NICE (2019) guidelines [13], this proposed pilot randomised controlled trial (RCT) will test the feasibility of implementing a RTW intervention for workers on long-term sickness absence due to poor mental wellbeing or with poor mental health as a comorbidity across SMEs and LEs across all sectors. This intervention has been named PROWORK (PROmoting a Sustainable and Healthy Return to WORK).

### Study aims and objectives

The primary aim of this study is to undertake a pilot RCT (PROWORK) to test the feasibility of implementing a RTW intervention across SMEs and large enterprises (LEs) from a range of sectors. Secondary aims include the evaluation of the primary research outcome to inform a fully powered definitive RTW trial and to conduct a full cost-utility analysis. The objectives of this pilot study are therefore as follows:

#### Objectives

1. Assess potential selection bias in control and intervention organisations as measured using participant characteristics at baseline.
2. Estimate retention of participants in the research evaluation at each follow-up timepoint across both control and intervention groups.
3. Assess implementation of intervention delivery, dose, fidelity engagement and adherence by workers and their line managers (or those with a responsibility for managing RTW).
4. Assess likely changes in the primary outcome (number of days until RTW part-time or full time).
5. To describe questionnaire data outcomes of interest (e.g. anxiety, depression; readiness to RTW)
6. To provide an early estimate of the costs, both healthcare and societal costs, in both intervention and control groups
7. Determine the willingness and readiness of employers and their workers to adopt the proposed intervention in a manualised format (written as an instruction manual) but that is flexible enough to meet individual and organisational needs in different settings.

## Methods/design

### Study design

**Fig. 1:**
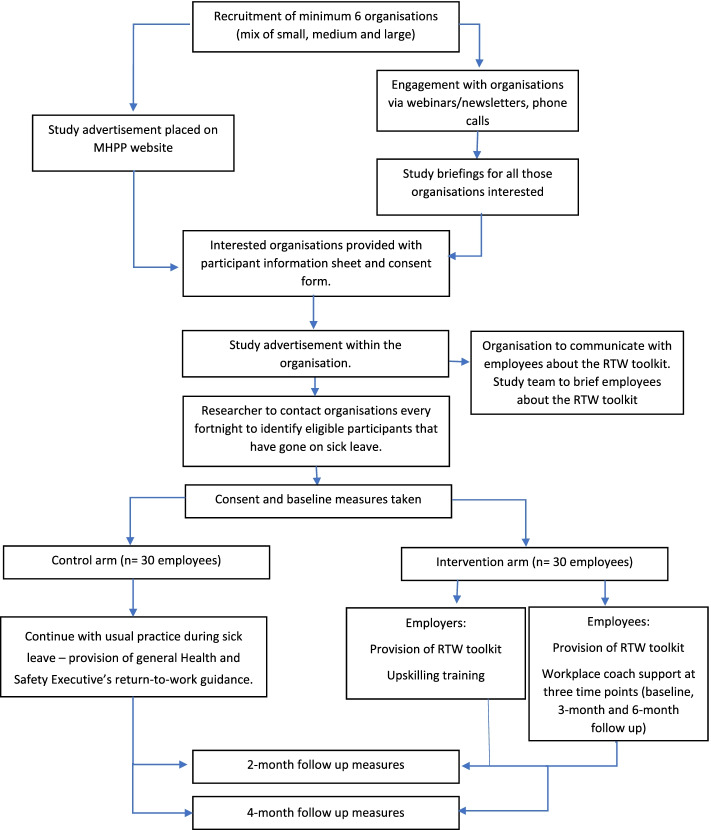
Study flow diagram

This is a feasibility randomised controlled trial with a 4-month intervention (called PROWORK) to be delivered to employees and managers in SMEs and LEs. Each of the organisations are the units of randomisation (the clusters), with data collected from individual workers (the participants). In addition, managers with a worker on LTSA will be eligible to participate. Although dyads of managers and workers can participate, this is not essential. This design overcomes the problem of contamination between the intervention and control arms and the problems associated with individually consenting and randomising workers to a trial where the employers may be managing the sick leave of 2 or more workers. A repeated measures design will be adopted, whereby worker participants will complete outcome measures at baseline, 2 months and 4 months, and managers will complete outcome measures at baseline and 4 months. Each organisation’s involvement in the trial is for 12 months. Observations and end-of-study interviews with workers and managers and informal monthly meetings with the organisation’s human resources representative will be conducted throughout the intervention period as part of a full process evaluation. Figure 1 shows the study flow diagram, and Table 1 indicates the schedule of enrolment, intervention and outcome measures. The project has been granted ethical approval by the Loughborough University Ethical Advisory Committee (reference 2020-1889-2041). The trial was preregistered in the ISRCTN registry on 15th December 2020 (10.1186/ISRCTN90032009).

**Table 1:** Standard Protocol Items: Recommendations for Interventional Trials (SPIRIT) diagram illustrating the design and timescales of the pilot PROWORK RCT

TIMEPOINT	Enrolment	Allocation	Baseline (0 month)	2-month follow-up	End of intervention (4-month)	End of study
**ENROLMENT**						
Organisation recruitment	x					
Eligibility screen	x					
Organisation informed consent	x					
Allocation		x				
Manager informed consent			x			
Worker informed consent			x			
**INTERVENTION**						
Worker and manager RTW toolkit			X	X	X	
Worker coaching sessions			X	X		
Manager training webinar			X			
Control group - usual practice			X	X	X	
**ASSESSMENTS**						
Organisational sickness policy data	X					
Organisational sickness report data			X			
Feasibility outcomes (recruitment)			X			
Feasibility outcomes (retention, compliance)					X	X
Process evaluation	X		X	X	X	X
Intervention resource used						X
Economic evaluation	X		X	X	X	X
**Worker measures**						
Demographics			X			
Days of sick leave			X	X	X	
Mental health			X	X	X	
Quality of life			X	X	X	
Healthcare resource used			X	X	X	
Return to work measures			X	X	X	
Workplace support and communication			X	X	X	
Work outcomes			X	X	X	
Intention to use toolkit			X	X		
Manager RTW actions				X	X	X
Interviews						X
**Employer measures**						
Demographics			X			
Mental health management experience			X			
RTW management experience			X			
RTW support for worker					X	X
Interviews						X

#### Setting

PROWORK is part of the Mental Health and Productivity Pilot (MHPP; https://mhpp.me/), a larger research programme which has been funded by the Midlands Engine (CPU 2640) to develop cost-effective and sustainable research to support good mental health at work, reduce stigma and increase productivity in businesses within the nine areas of the Midlands region in the UK. This is the UK’s largest regional economy outside London (£246 bn a year), and it is home to a sixth (11 million) of the UK’s population, concentrating a mixture of organisations ranging from advanced technology to transport, research and manufacturing. Therefore, this study will take place in a wide range of organisational settings across the Midlands and will involve small (10 to 49 workers), medium (50 to 249 workers) and large (over 250 workers) enterprises.

#### Sample size

Recommendations for feasibility pilot RCT sample sizes were followed. Guidance for pilot clinical trials [16] recommends recruiting a minimum of 4 clusters per arm. Additionally, feasibility RCTs are recommended to recruit between 24 [17] and 50 [18] participants per arm, consistent with the median sample size found in pilot RCTs [19]. This is in line with the guidance from the National Institute for Health Research (NIHR), which indicates that a sample size of 30 is appropriate to answer the questions posed by a feasibility trial [20]. To match these recommendations, we aim to recruit at least 8 organisations of all sizes (small, medium, large) (4 controls and interventions) and 30 participants, including workers and managers, in the intervention group and the 30 in the control group. Participating organisations will be recruited over a 6-month period; the recruitment of workers and their managers will continue for 18 months.

#### Organisation recruitment and inclusion criteria

Small, medium and large organisations from across all sectors within the Midlands region, UK, will be invited to participate in PROWORK. Organisations will be recruited via webinars, phone calls, newsletters, emails and social media. The study will also be advertised through the Mental Health Productivity Pilot (MHPP) programme’s network, where organisations can fill out an expression of interest form. Organisations who express an interest in the study will be asked to provide data on the prevalence of their long-term sickness absence over the last 12 months along with a description of their RTW policy and procedure to cross-check for any overlaps or contradictions to PROWORK. Those organisations with conflicting policies that overlap PROWORK will not be eligible for the study. Eligible organisations are small, medium and large organisations with at least 2, 4 and 6 workers, respectively, on long-term sick leave over the past 12 months and with no contradictory or overlapping RTW polices.

Organisations will be asked to promote the study to potential participants (workers on long-term sick leave and their managers) through direct email and newsletters. To ensure the organisations’ compliance and timely recruitment of participants, monthly meetings will be arranged between the research team and the appointed organisation leads.

#### Participant recruitment, inclusion criteria and consent

Within participating organisations, workers (over 18 years of age) that go on sick leave for at least 8 days (or from issue of fit note) and up to 8 weeks (50 days) and/or the person managing their sickness absence are eligible to participate. Sickness absence must be associated with either poor mental wellbeing or where poor mental wellbeing may be a comorbidity [14]. Once the worker on long-term sick leave is identified by the organisation, both the worker, and their manager, will be sent a detailed information sheet about the study so that they can make an informed decision about their participation. Those interested in taking part will contact the research team directly either by phone or email, at which stage they will be consented by the research team.

Workers on long-term sick leave will not be eligible to participate in PROWORK if they are on sick leave (a) with a psychotic episode such as schizophrenia, or with substance abuse, (b) whilst under formal investigation for misconduct or in the formal process of disciplinary action, (c) due to cancer and signed off work for at least 6 months and (d) due to a neurological condition (e.g. multiple sclerosis, Parkinson, dementia).

#### Allocation to intervention

In this pilot RCT, organisations will be stratified by size. To avoid study contamination, organisations will be randomised into the intervention or active control group after baseline measures are taken using an allocation ratio of 1:1. Randomisation will be carried out by computer-generated randomisation stratified by organisational size (small, medium or large).

Consented workers will be allocated to either the intervention or active control group based on the randomisation of their participating organisation. Baseline data will be collected from each participant prior to either being given the RTW toolkit (intervention group participant) or supporting information (active control group participant). The participating workers and employers will not be blinded in group allocation.

#### Intervention

This RTW intervention uses a multicomponent approach grounded on behavioural change techniques (BCTs) to promote early and positive communication between the worker and the person managing their sick leave. The overall aim is to reduce the number of days on long-term sickness and enable a successful RTW. The intervention comprises of 2 toolkits — an employer and worker RTW toolkit. Both toolkits are self-led interventions used by the employer (or manager) and the worker themselves. The guidance and resources in the toolkits for the worker and the employer mirror each other to ensure both receive the same messages and to encourage transparency.

**Fig. 2:**
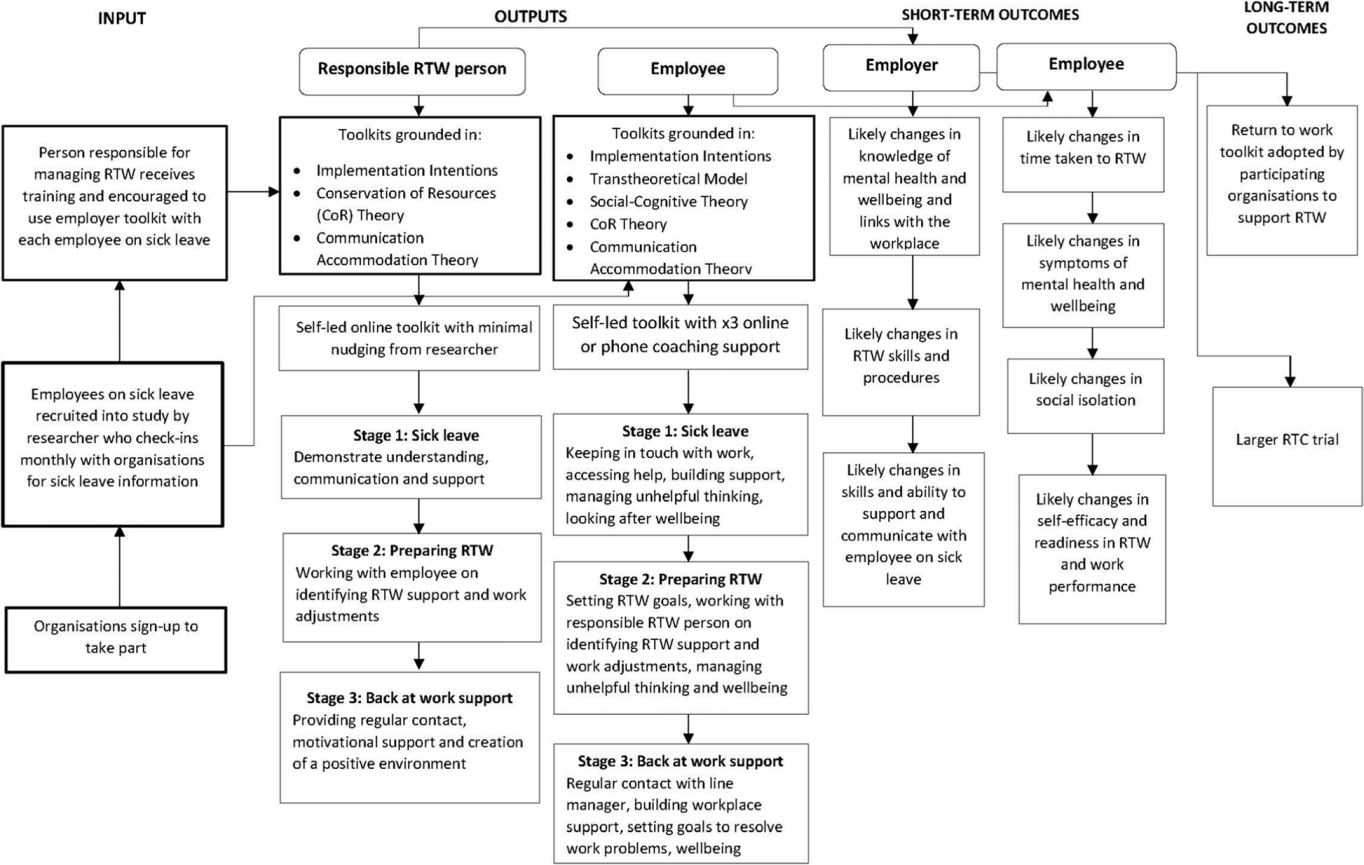
PROWORK logic model

Once participants have been consented into the intervention, they will be able to access the toolkits through a secured website with a code provided by the research team. The toolkits include three step-by-step approaches to be used at different stages of the workers’ RTW process: step 1 managing initial sick leave, step 2 preparing to RTW and step 3 managing back at work. Additionally, the worker toolkit is supported by a coaching component, and the employer toolkit is supported by an upskilling training webinar session. Both toolkits have been cocreated with workers with mental health and RTW experience and managers and employers from SMEs and LEs. Both toolkits have also been developed with input from the charity Mind as well as evidence from the scientific literature and best practice guidelines for the UK (NICE, 2019). PROWORK toolkits are grounded in the implementation intentions theory [21], conservation of resources (CoR) theory [22] and communication accommodation theory [23]. In addition, the worker toolkit is also grounded in the implementation intentions [24], transtheoretical model of change [25] and the socio-cognitive theory [26]. The cognitive behavioural elements (e.g. unhelpful thinking worksheet) in the toolkit are also informed by principles of problem-solving and cognitive behavioural approaches [27–29]. Table 2 provides a description of each intervention components, whilst Fig. 2 outlines the logic model.

**Table 2:** Description of intervention components

Intervention component	Description	
**Management of sickness absence and RTW**	**Worker toolkit** - Education ○ How and when to use the toolkit ○ Sick leave and wellbeing - Step-by-step guidance of how to self-manage ○ Sickness absence ○ Preparing to RTW ○ Being back at work - How to build confidence in managing mental health? - How to prepare to RTW? - Knowledge of different signs of low mental wellbeing - Where to find support to improve my mental health? - What to do for a sustainable RTW? **Barrier identification** - Is there anything that might stop you from using the toolkit? - Is there anything that might stop you from having a successful RTW? - How can you overcome these challenges? **Behavioural change techniques** - Introduction to step-by-step techniques to improve health and build confidence to RTW - Introduction to styles of thinking and management of unhelpful thinking - Introduction to SMART goals - Using checklists and activities to track changes and plan ahead	**Employer toolkit** - Education ○ How and when to use the toolkit? ○ How to apply a person-centric approach? ○ Sick leave and mental wellbeing - Step-by-step guidance of how to manage ○ Managing sickness absence ○ Preparing for worker’s RTW ○ Managing the worker back at work - How to be in the right mindset to manage the RTW well? - How to develop positive communication skills? - Knowledge of different signs of low mental wellbeing - Where to find support to manage worker’s RTW? - What to do for a sustainable RTW? **Barrier identification** - Is there anything that might stop you from using the toolkit? - Is there anything that might prevent you from applying positive communication techniques? - How can you overcome these challenges? **Behavioural change techniques** - Introduction to step-by-step techniques to build confidence and manage RTW positively - Introduction to positive communication techniques - Introduction to goal setting - Using checklists and activities to track changes and plan ahead
**1-h worker coaching session**	- Three workplace coaching sessions based on goal setting and problem-solving skills to support the worker during their sick leave and RTW - Goal setting and revision of previous and future goals and their outcomes - Identification of challenges and facilitators of each of the goals - Revision of activities and planning of the next steps	
**20-min employer training webinar**	- Initial training module to inform the person managing the sick leave and RTW of a worker of how to have conversations about mental health at work and help the worker feel comfortable and confident about having those conversations	
**Prompts**	- Prompts offered by researcher to encourage worker and employer to use the toolkit	
**Intervention checklists**	- Completion of checklists at each of the sickness absence and RTW stages - Rate confidence to RTW - Rate mental wellbeing	
	- Researcher to ask participants to send their completed checklists for compliance	
**Group messaging**	- To be used as a platform to contact employees to record their RTW date

### Workers

Workers on sick leave will receive the intervention until RTW or 4 months (last follow-up) from when the worker has completed baseline measures. PROWORK includes a step-by-step action-oriented toolkit that provides guidance and support from initial absence to post RTW. Three workplace coaching sessions based on goal setting and problem-solving will be offered to worker by a trained project researcher over the phone. These will be delivered at three timepoints throughout the intervention: (1) at the start, to support making contact, to coach the participant in the use of the resources, to build a relationship with the participant and to direct the participant to external resources available in the toolkit; (2) at 2 months to coach them for preparation to RTW, or if they have returned to work, and then to coach them in adjusting back to work; and (3) at 3 months to either coach them for preparation to RTW or if they have returned to work and then to coach them in adjusting back to work. The coaching sessions aim to motivate and support workers to use the RTW toolkit, to help them with any activities they might feel stuck with and to discuss their action plan to avoid any relapse. During these sessions, and as part of the process evaluation, the workplace coach will ask about what resources are the worker finding more useful and what are the benefits of using this toolkit.

To reinforce the message of having regular communication with the workplace whilst on sick leave, the worker will have the option to contact the workplace coach (i.e. project researcher) when needed either by email or phone call.

### Employers

Employers will have access to a RTW training video at the start of the intervention study, prior to recruiting workers on sick leave. The key workplace person (e.g. the line manager) responsible for managing a worker on sick leave will receive an employer version of the toolkit and provided with advice on when and how to use it with a worker. For those individuals who are managing more than one worker on long-term sick leave, they will receive an email from the researcher at the start of each worker on long-term sick leave, to remind them to use the toolkit (once they had done the webinar training).

#### Active control group

To compare the effects of the intervention against usual practice, organisations allocated into the control group will be asked to continue with their usual procedures, and no training or guidance will be offered. Participants in the control group will be asked to complete the same measurements as those in the intervention group and at the same timepoints. At the end of the PROWORK study, organisations in the control group will receive a report evaluating their current RTW policies and procedures, access to the line manager training and hard copies of both toolkits.

#### Outcome measures

Primary and secondary outcome measures will be collected at baseline, 2 months and 4 months. Outcome measures will be collected for both workers and managers. Outcome measures have been grouped as process outcomes and research outcome measures (see below), and they will be collected using Qualtrics; data and personal details will be stored according to the General Data Protection Regulation (GDPR, 2016/679) and research governance guidelines. A summary of the outcome measures at the various time points is provided in Table 3. Qualitative interview data will be collected either over the phone or using online conferencing facilities. Both full-return (defined for this study as working the same days or hours per week as before sickness absence in an identical or equivalent role for at least 4 weeks) and partial RTW (defined as working any number of hours in any role) data will be collected via text messages.

**Table 3:** Schedule of process and research outcome measures

Measured outcome	Assessment tool	Pre-randomisation	Baseline	Month 1	Month 2	Month 3	Month 4
Process outcomes							
Long-term sickness absence data for the past 12 months	Reports review	**X**					
Frameworks (sickness absence policy, return-to-work policy, mental health training and support)	Policy review	**X**					
Number of workers on (a) sick leave (≥ 8 days) and their reasons and (b) that employers have contacted to take part in the study (until the end of the study)	Checklist		**X**	**X**	**X**	**X**	**X**
Number of persons responsible for managing the sick leave and return to work (a) consenting to take part and (b) attending the training	Checklist	**X**					
Toolkit use	Volute records and interview		**X**				**X**
Feasibility and acceptability of intervention	Interview						**X**
Toolkit use	Interview						**X**
Research outcome worker							
Workers sick leave	Self-reported number of days						**x**
Depression	Patient Health Questionnaire- 9		**X**		**X**		**X**
Anxiety	General Anxiety Disorder-7		**X**		**X**		**X**
Sick leave duration	Number of days on sick leave		**X**		**X**		**X**
Self-efficacy to RTW	11-item Return-to-work Self-Efficacy Scale		**X**		**X**		**X**
Readiness to RTW	13-item Readiness to Return to work		**X**		**X**		**X**
Readiness to stay at work	9-item Readiness to Stay at Work Scale		**X**		**X**		**X**
Workplace communication	6-item Workplace Health Communication Scale		**X**		**X**		**X**
Work productivity	Work Productivity and Activity Impairment: General Health v2.0 (WPAI:GH)				**X**		**X**
Job satisfaction	A 1-item job satisfaction scale		**X**		**X**		**X**
Intention to use toolkit	4-item Toolkit Use at coaching session		**X**	**X**	**X**		
Health related quality of life	Euro-QOL — five-dimension scale		**X**		**X**		**X**
Demographics	Self-reported questionnaire		**X**				
RTW actions	Adaptation of the line manager behaviour questionnaire		**X**		**X**		**X**
Research outcomes employer							
Mental health experience	1-item questionnaire		**X**				
Sickness absence management training	1-item questionnaire		**X**				
LTSA and RTW management experience	1-item questionnaire		**X**				
Demographics	Self-reported questionnaire		**X**				
Actions to support RTW	The measure for supervisors to support return to work (SSRW)						**X**

#### Trial process-related outcome

Organisational data collection pre-randomisation is as follows:

1. Summary of long-term sickness absence data for the past 12 months (only total numbers and % by reasons)
2. Organisation size and sector
3. Copies of sickness absence policy and frameworks
4. Copies of the work policy and frameworks
5. Details on mental health training and support available for managers

Organisational data collection post-randomisation and the duration of the study are as follows:

1. Number of workers on sick leave (≥ 8 days) and their reasons
2. Number of workers that employers have contacted to take part in the study
3. Number of workers consenting to take part
4. Number of workers using the toolkit (data collected at interviews and website use)
5. Number of persons responsible for managing the sick leave and RTW of workers consenting to take part
6. Number of persons responsible for managing the sick leave and RTW of workers attending the training
7. Number of persons responsible for managing the sick leave and RTW of workers using the employer toolkit (data collected at interviews and website use)

##### Coverage (recruitment and attrition)

Where possible, data on reach of study participation advertisement, expressions of interests, recruitment, participation and drop-out for all participants.

##### Toolkit use/adoption

The PROWORK website has an in-built Google Analytics system, which will provide metrics information such as number of visits to the site per day, behaviour of viewers once on the website (i.e. which pages they visited), where most of the traffic to the toolkit website came from, and the percentage of returning participants.

### Trial feasibility and acceptability-related outcomes

We will collect data on the following:

1. Willingness of organisations to take part (baseline) and retention through follow-up with reach, uptake and completion as primary endpoints.
2. Proportion of workers on sick leave referred to pilot study (> 50% green for main trial to go ahead, 30–50% amber; < 30% red).
3. Proportion of workers and line manager participating in both control and intervention groups (worker: > 50% green, 30–50% amber; < 30% red)
4. For intervention worker participants, short worker interviews will be conducted at each coaching session (at intervention start, 2 months and 3 months) to explore use of the toolkit.
5. At 4 months (end of intervention), an interview will be conducted with both worker participants and line manager/RTW contacts to ask them about their engagement, usage and the effectiveness of the toolkit. Information regarding barriers and facilitators to implementation, functionality and interest in on-going usage will also be assessed.
6. At the end of 12-month study period, organisational stakeholder will be interviewed to explore their perceived benefits of the intervention (engagement, usage, functionality and the effectiveness of the toolkit), as well as barriers/facilitators to implementation, interest in on-going usage and whether the intervention could form part of any workplace RTW policies.
7. Costs associated with toolkit website build and delivery, training delivery, coaching sessions and other associated costs will be collected.

### Trial research-related outcomes

#### Primary worker measures (at baseline, 2 months and 4 months)

The primary research outcome of the intervention will be assessed monthly, after randomisation at the worksite level using 1:1 ratio approach. For the primary research outcome, we will contact (text message) the workers from the control and intervention group on monthly basis and will ask them to report if they are still on sick leave or if they are planning to RTW. For those who have returned to work, we will ask them for the date of their first day back at work (and whether it is a partial or full return). The RTW date will also be collected from the organisational records. Secondary outcome measures of the intervention will be assessed at 3 timepoints (within 2 weeks of consenting, at 2 months and at 4 months).

Number of days of sick leave will be recorded from the employer and from the worker (self-report). Self-report data will be collected at baseline at 2 months and at 4 months (from control and intervention participants) using an online survey. Participants will be asked the following questions: (a) are you still on sick leave? If yes, do you have an RTW date? If not on sick leave, when did you go back to work? (b) How many hours are you currently working? (c) Is this the same as before your sick leave? Those who have returned to work will be asked to report their first day back at work (and whether it is a partial or full return). The last data collected will be at 4-month post-randomisation.

#### Secondary worker measures at baseline, 2 months and 4 months

1. *Self-report mental health:* the 9-item Patient Health Questionnaire (PHQ-9) [30] and the 7-item General Anxiety Disorder (GAD-7) [31] will be used to measure depression and anxiety, respectively. The PHQ-9 is used by GPs and practitioners involved in the Improving Access to Psychological Therapies (IAPT) initiative, providing an opportunity to compare the outcomes of this study directly with routine care. Both measures accurately reflect improvement and worsening of symptoms of depression and anxiety.
2. *Return to work measures*: Expectations about length of sick leave will be asked using one question from Aasdahl et al. (2018) [32]: “For how long do you believe you will be on sick leave from today?” with 6 response options “not at all”, “less than 1 month”, “1–2 months”, “2–4 months”, “4–10 months” and “more than 10 months”. The Lagerveld et al. (2010) [33] 11-item Return-to-work Self-Efficacy Scale and the Franche et al (2007) [34] 13-item Readiness to Return to work will be used to assess confidence and readiness to RTW. For those who have returned to work, the 9-item Readiness to Stay at Work Scale [34] will be used.
3. *Workplace support and communication*: 6-item Workplace Health Communication Scale (Yarker et al., no date) will be used to assess quality of communication between the worker, employer and organisation.
4. *Work outcomes*: For those who have returned to work at 2 months and 4 months, work productivity will be measured using the Work Productivity and Activity Impairment: General Health v2.0 (WPAI:GH) [35]. The WPAI:GH yields 4 types of scores: “absenteeism”, “presenteeism”, “work productivity loss” and “activity impairment”. A 1-item job satisfaction scale will be used to assess satisfaction [36].
5. *Intention to use toolkit*: A 4-item Toolkit Use (Yarker et al., no date) will be used to assess motivation and engagement for those in the intervention group. These questions will be asked at each coaching session.
6. *Quality of life*: Health-related quality of life will be assessed using the EQ5D-5L [37].
7. *Demographics and other measures*: Basic demographic information for each participant including their date of birth, ethnicity and highest level of education will be collected. The average wage for each worker will be identified using the UK Standard Occupational Classification coding and annual earnings data for each job type. Workers will also be asked if they are the main wage earner. Information on medical diagnosis of health conditions, prescribed medication use and other current therapeutic treatments for mental health will be collected (adapted from Peveler et al., 2005 [38];).
8. Participants will be asked what actions their workplace contact (i.e. person responsible for managing their return) carried out to support their RTW. The line manager behaviour questionnaire [39, 40] will be adapted for this purpose.

### Secondary employer measures at baseline

1. *Mental health and RTW experience*: Those with responsibility for RTW will be asked about their experience with mental health: “how much experience you have with mental health either yourself or through a close friend or family member?” with five response options: “none at all”, “a little”, “some”, “quite a lot” and “ a great deal” (1 item, Yarker et al., no date); and 2-item training question “indicates how much formal training you have had in (a) managing mental health of others and (b) training in sickness absence management and RTW” with five response options: “none at all”, “a little”, “some”, “quite a lot” and “a great deal” (1 item, Yarker et al., no date); and a 1-item question of long-term sickness absence and RTW management experience: “We would like to know how much experience you have with managing long-term sick leave and RTW in the past 12 months” with five response options: “none at all”, “1 worker only”, “2–3 workers”, “4–5 workers” and “more than 5 workers”.
2. *Demographics*: Data on age, gender, ethnicity, job role and tenure will be collected.

#### Secondary employer measures at 4 months

Participants will also be asked what actions they carried out to support the return of their worker. The measure for supervisors to support return to work (SSRW) [39, 40] will be adapted for this purpose.

#### Process evaluation analysis

A detailed process evaluation informed by the Implementation Outcome Framework (IOF) [41, 42] will be conducted. This framework includes eight implementation outcomes (acceptability, adoption, appropriateness, feasibility, fidelity, implementation cost, coverage and sustainability). In addition, the Theoretical Domains Framework (TDF) [43, 44], a widely used framework in behaviour change and implementation research, will also inform the process outcome data collection. Use of the TDF allows an in-depth exploration of the barriers and facilitators of implementing the trial. The data collection methods and its process outcomes outlined below capture the process outcome information for the IOF and TDF.

End of study semi-structured interview schedules will be developed to explore overall participant’s experiences using PROWORK during their sickness absence and RTW. Similar interview schedules will be created to explore the main barriers and facilitators to engaging and implementing PROWORK at the organisational level. These will be supplemented with researcher notes, including thoughts and observations about the organisation’s procedures and implementation approach and notes from the monthly calls between the research team and the organisation’s HR contact. Collectively, these will provide information on the acceptability of the trial procedures including randomisation, the measurement instruments and the overall acceptability of the intervention. Interviews and monthly calls will be conducted by a conferencing platform, so they can be recorded and transcribed verbatim. Short employer interviews (e.g. with HR, health and safety manager) with intervention and control sites at monthly intervals will be conducted to explore any changes to policies or processes that may impact the study. Questions around study participation (e.g. identifying workers on sick leave, sending out study information) will also be explored, and formal notes will be taken as part of the process evaluation. A detailed plan of the measures and methodologies used in the process evaluation is described below.

### Process outcomes

1. The number of organisations agreeing to participate in the trial will be summarised in terms of their size, sector, sick leave and RTW polices and number of workers who were on long-term sick leave in the past 12 months prior to the start of the study.
2. The number of worker participants identified on long-term sick leave and the number recruited into the study will be reported, along with the number of participants followed up at each timepoint. Withdrawals (and where possible, reasons for withdrawals) will be reported.
3. Difference in recruitment uptake rate and follow-up rates at each time point will be compared between the intervention and control arms.
4. As organisations of different sizes are taking part, it is likely there will be some imbalance between participants in each treatment arms on one or more baseline characteristics. Baseline comparisons will be carried out to detect any substantial differences between participants recruited from the control and intervention arms. This will be done by scrutinising the baseline data for any serious imbalances in observable baseline variables and the trends of the imbalance if any. The recruitment rates will also be estimated and compared between the control and intervention arms. The size of any imbalances will be examined, in addition to evidence of systematic selection bias in the types of patients being recruited in control versus intervention arms
5. Key baseline characteristic will be compared between those participants followed up and those lost to follow-up at each timepoint.
6. Intervention fidelity will be assessed by the log in and downloads of the toolkits. Successful adherence is defined as at least 60% download of the total toolkit by workers and employers (i.e. those with a responsibility for managing RTW) and at least 60% completion of the activities/checklist in total.
7. Following the guidance from the COREQ-32 checklist [45], qualitative interview data for the process evaluation will be recorded, transcribed verbatim and coded following the principles of thematic analysis [46].
8. The Theoretical Domains Framework [43], and the normalisation process theory [47], will be used to guide thematic analysis of the qualitative data. The findings will be supplemented with observations made by the researchers throughout the implementation of the intervention. Collectively, these will provide information on the acceptability of the trial measurements and the intervention.
9. Survey data for the process evaluation will be summarised using means, standard deviations, medians and ranges for continuous variables and counts and percentages for categorical variables.
10. The pilot data will provide information on the parameters needed for a realistic sample size calculation (mean and standard deviation) for a future, main cluster RCT.

#### Participant appreciation

As a thank you for participating in the pilot trial, participants will have the chance to win a £50 gift voucher for every survey they complete during the baseline and follow-up measures to encourage participation.

#### Cost analysis

The cost analysis will be exploratory, with the aim to inform the design of a full cost-utility analysis alongside a future main trial. Data on costs will be sought from all participants, and results will be presented taking into account worker-incurred costs and productivity losses. Analyses will be mainly descriptive, and all costs and outcomes will be summarised using means and 95% confidence intervals.

1. Healthcare resource used will be collected using self-completed questionnaires at baseline, 2 and 4 months, with a recall period of 2 months in each. Questions will ask workers to recall GP consultations, visits to healthcare professionals, outpatient appointments, investigations or treatments and inpatient stays related to the index condition (adapted from Peveler et al., 2005 [38]). Participants will be asked to distinguish between National Health Service (NHS) and private practice visits.
2. Resource used for the intervention will be directly recorded and costs attached, staff time (e.g. coaching sessions, training sessions), materials (posters, flyers, referral forms, website set-up and maintenance, toolkit printing) and training sessions.
3. Costs will take into account both absenteeism and presenteeism and will utilise self-report data on employment status, occupation and time off work and reduced productivity at work (presenteeism).
4. All workers will be asked to complete the 5-level version of the EuroQoL-5DL (EQ-5DL) (1990) [48] (questionnaire at baseline, 2 months and 4 months) in order for the quality-adjusted life years (QALYs) over the 6-month time period to be calculated for each participant. The QALYs combine information on health-related quality of life and survival.
5. Productivity costs will be calculated using data collected on the absence from the number of days taken to RTW.
6. Using the human-capital approach (which assumes that the value of lost work is equal to the amount of resources an individual would have been paid to do that work), the self-reported days of absence will be multiplied by the respondent-specific wage rate.

### Statistical data analysis

This study will be analysed according to the Consolidation Standards of Reporting Trials (CONSORT) statement for cluster RCTs [49]. As this is a feasibility study, main analysis will include descriptive statistics (mean, SDs and medians). Data will be analysed using IBM SPSS statistics. Data analysis will be performed after the last trial participant has completed their 4-month post-intervention. A baseline table (descriptive statistics and frequencies) will compare the demographic and clinical characteristics including gender, age, education, number of days on sick leave, mental health status, readiness, intention and self-efficacy to RTW, work support, communication, performance at work, sleep and physical activity, between the 2 arms. As this is a pilot trial, no emphasis will be put on the *p*-values, as the main purpose of the analysis is to calculate confidence intervals (CI). Statistical analysis will be carried out on an intention to treat basis with missing outcome data being imputed using multiple imputation. To explore the extent and patterns of missing outcome data, we will report the proportion of missing values per item, proportion of participants who complete all items on the questionnaire and the proportion of respondents who answer at least 50% of the items in a scale. The proportion of missing data will also be reported for the other key outcomes and compared between the participants from intervention and control practices. The point estimate of the proportion and its 95% confidence interval (CI) will be provided for the primary research outcome, i.e. number of days taken to RTW (partial or full) for the intervention and the control arms. Key baseline characteristic will be compared between those participants followed up and those lost to follow-up at each timepoint.

#### Data management and research governance

All data will be anonymised and entered into a secure database and only accessible by trial staff and authorised personnel. The study will comply with the Data Protection Act which requires data to be anonymised as soon as it is practical to do so. Personal data will be processed on the public task basis under the General Data Protection Regulation (GDPR). The study will be sponsored by Loughborough University. An independent Trial Steering Committee (TSC) will be created to oversee the evolution of the study, and they will meet every 4 months. Due to the low-risk nature of this study, no adverse events are anticipated to occur; however, should any arise, the TSC and the Loughborough University guidelines will be consulted for the managing and reporting of any intervention-related adverse events.

## Discussion

This article describes the protocol of a novel study testing the feasibility and acceptability of conducting and evaluating a multicomponent RTW intervention. Evidence-based RTW interventions for workers on long-term sick leave due to poor mental wellbeing or where poor mental health is a comorbidity in the UK are scarce. Yet, robust evidence from systematic reviews and meta-analysis [10–12] highlights the benefits of work-related interventions to improve workers’ mental health and RTW experience. Positive communication and regular contact with the workplace are key factors to the success of such interventions and can lead to a significant reduction of the sickness absence [11], particularly if combined with an understanding of workers’ expectations in the early phase of their sickness absence [11].

Poor mental wellbeing increasingly accounts for a significant proportion of long-term sickness absence from work [1], and those who are absent for 6 months or longer have less than a 50% chance of ever returning to employment [2]. Common mental disorders are also long-lasting predictors of duration and recurrence of sickness absence, reduced productivity, work disability, and early retirement [50]. Therefore, early intervention to support a worker back to work is vital for the worker, as work provides income, structure and social connections to an individual’s life and the employer (e.g. reduced turnover, recruitment costs, retention of knowledge and culture of wellbeing).

This study protocol describes a 4-month intervention programme to promote positive communication for a sustainable RTW following a period of long-term sickness absence, with evaluation measures taken at baseline, 2 months and 4 months. Development of this RTW intervention is grounded in BCTs [24–26], mirrors conversation techniques and training for employers, to underpin a change in sickness absence management and RTW practices that promote a positive and sustainable RTW experience.

One of the main challenges will be associated to the recruitment of workers on long-term sick leave due to poor mental wellbeing. Although early communication with workers on long-term sick leave is encourage by the official guidelines [13], organisations might not feel comfortable in doing so and/or they may not want to overload their workers with extra information. To minimise risks to recruitment, an evidence-based strategy has been developed informed by the latest scientific RTW literature [10–12] and the official government guidelines [13].

The current NICE (2019) guidelines [13] on sickness absence agree that communication of the organisation’s policies and procedures to their workers and having a supportive culture that promotes health and wellbeing are important for organisations of all sizes. However, these guidelines do not offer step-by-step best practice guidance to manage a worker’s sickness absence and RTW, and smaller organisations might not even have standard policies and procedures in place. The need for more, and effective, RTW interventions targeting people with poor mental wellbeing on long-term sickness by encouraging a healthy and sustainable RTW is clearly recognised by the public bodies [13] and organisations of all sizes and sectors [1]. Whilst there is evidence of its efficacy at helping people RTW, this is not UK based [10–12]. Providing the complexity of this intervention and the target population, the Theoretical Domains Framework [43, 44] used to evaluate this trial, will offer an insight from the experiences at the organisational and individual that will allow to effectively tailor the PROWORK components to suit the needs of all-size organisations. Therefore, this pilot RCT will provide essential preliminary data about the feasibility of implementing a RTW intervention to support those experiencing poor mental wellbeing whilst on sick leave and with a comprehensive understanding of whether a full randomised control trial is viable in UK small and medium enterprises and large organisations.

## Data Availability

The datasets generated during the study will be available from the corresponding author on reasonable request.

## Abbreviations

RTW: Return to work
RCT: Randomised controlled trial
SMES: Small- and medium-sized enterprises
LEs: Large enterprises
CONSORT: Consolidation Standards of Reporting Trials
SPIRIT: Standard Protocol Items: Recommendations for Interventional Trials
GDPR: General Data Protection Regulation
LTSA: Long-term sickness absence
WHO: World Health Organisation
TSC: Trial Steering Committee
UK: United Kingdom
NICE: National Institute for Health and Care Excellence
MHPP: Mental Health and Productivity Pilot
PROWORK: Promoting a sustainable return to work
CoR: Conservation of resources
PHQ: Patient Health Questionnaire
GAD: General Anxiety Disorder
IAPT: Improving Access to Psychological Therapies
WPAI:GH: Work Productivity and Activity impairment: General Health
SSEW: Supervisors to support return to work
IOF: Implementation Outcome Framework
TDF: Theoretical Domains Framework
HR: Human resources
QALYs: Quality-adjusted life years
CI: Confidence interval
